# Refractory phenotype of *Aspergillus*-sensitized asthma with bronchiectasis and allergic bronchopulmonary aspergillosis

**DOI:** 10.1016/j.jacig.2024.100364

**Published:** 2024-11-01

**Authors:** Natsuko Nomura, Hisako Matsumoto, Koichiro Asano, Yusuke Hayashi, Akihito Yokoyama, Yoshihiro Nishimura, Naozumi Hashimoto, Takuro Sakagami, Koichi Fukunaga, Nobuyuki Hizawa, Akira Yamasaki, Hiroyuki Nagase, Noboru Hattori, Mitsuko Kondo, Norihiro Harada, Hisatoshi Sugiura, Mari Miki, Tomoki Kimura, Mikio Toyoshima, Osamu Matsuno, Hidefumi Koh, Toshiyuki Kita, Hiromi Tomioka, Keisuke Tomii, Hisashi Ohnishi, Shohei Takata, Kazunori Tobino, Shiro Imokawa, Hironobu Sunadome, Tadao Nagasaki, Tsuyoshi Oguma, Naoya Tanabe, Toyohiro Hirai, BEXAS study, Miho Ikeda, Kayoko Okamura, Hisashi Ohnishi, Junko Terada-Hirashima, Masayuki Hojo, Sumito Isogai, Kazuyoshi Imaizumi, Takahiro Horiguchi, Ryosuke Hirano, Masaki Fujita, Mikio Toyoshima, Tomoyuki Fujisawa, Takafumi Suda, Yoichi Takaki, Naoko Higaki, Shintaro Miyamoto, Taku Nakashima, Hiroshi Iwamoto, Noboru Hattori, Koji Mikami, Toshiyuki Minami, Ryo Takahashi, Takashi Kijima, Kazunori Tobino, Makoto Hoshino, Shiro Imokawa, Taisuke Tsuji, Noriya Hiraoka, Tatsuyoshi Ikeue, Takakazu Sugita, Naomi Kunichika, Shinya Tomari, Yasumi Okochi, Naoko Mato, Koichi Hagiwara, Kunio Dobashi, Yasuyuki Taooka, Norihiro Harada, Kentaro Machida, Hiromasa Inoue, Takae Tanosaki, Katsunori Masaki, Koichi Fukunaga, Akiko Sano, Takashi Iwanaga, Yuji Higashimoto, Yuji Tohda, Masataka Matsumoto, Kiyonobu Takatsuki, Kazuma Nagata, Ryo Tachikawa, Keisuke Tomii, Masahiro Kaneko, Hiromi Tomioka, Tatsuya Nagano, Yoshihiro Nishimura, Mayuka Yamane, Akihito Yokoyama, Chieko Yoshida, Takuro Sakagami, Yurie Seto, Yoshiko Kaneko, Koichi Takayama, Yusuke Hayashi, Satoru Terada, Kenta Nishi, Hironobu Sunadome, Tadao Nagasaki, Tsuyoshi Oguma, Naoya Tanabe, Toyohiro Hirai, Natsuko Nomura, Hisako Matsumoto, Tomoko Tajiri, Akio Niimi, Saya Nakamura, Keiko Wakahara, Naozumi Hashimoto, Takefumi Ito, Takako F. Nakano, Takafumi Yamashita, Shohei Takata, Yoshihiro Seri, Yasuyuki Mizumori, Hiroaki Tsukamoto, Ryogo Kagami, Yasuharu Nakahara, Tetsuji Kawamura, Yukio Ishii, Toshiyuki Kita, Kouko Hidaka, Toru Kadowaki, Masayoshi Minakuchi, Tomomasa Tsuboi, Shinji Tamaki, Takanori Matsuki, Hiroshi Kida, Mari Miki, Katsuyuki Tomita, Takashi Abe, Joe Shindoh, Akihiko Taniguchi, Nobuaki Miyahara, Masato Azuma, Mikio Kataoka, Osamu Matsuno, Haruhiko Ogawa, Takeshi Matsumoto, Kensaku Aihara, Kazuyuki Nakagome, Makoto Nagata, Satsuki Miyajima, Kentaro Hashimoto, Tetsuhiro Shiota, Masafumi Yamaguchi, Yasutaka Nakano, Kojiro Otsuka, Masanori Yasuo, Masayuki Hanaoka, Takashi Yamada, Toshihiro Shirai, Yoshinobu Iwasaki, Masamichi Mineshita, Takahiro Tsuburai, Yuko Komase, Hidefumi Koh, Koichi Hasegawa, Hideo Kita, Hiroyuki Nagase, Koji Murakami, Hisatoshi Sugiura, Masakazu Ichinose, Tomoko Kutsuzawa, Tsuyoshi Oguma, Jun Tanaka, Koichiro Asano, Yuta Kono, Shinji Abe, Morio Nakamura, Mami Orimo, Etsuko Tagaya, Mitsuko Kondo, Toshiaki Matsuda, Tomoki Kimura, Tomoya Harada, Akira Yamasaki, Hirokazu Taniguchi, Hiroaki Iijima, Hiroki Kawabata, Kazuhiro Yatera, Hironori Masuko, Yuko Morishima, Nobuyuki Hizawa, Masanori Nakanishi, Nobuyuki Yamamoto, Sumito Inoue, Kazuki Hamada, Yoshikazu Yamaji, Tsunahiko Hirano, Kazuto Matsunaga, Yoko Sato

**Affiliations:** a Department of Respiratory Medicine, Kyoto University Graduate School of Medicine, Kyoto, Japan; b Department of Respiratory Medicine and Allergology, Kindai University Faculty of Medicine, Osaka, Japan; c Division of Pulmonary Medicine, Department of Medicine, Tokai University School of Medicine, Kanagawa, Japan; d Department of Respiratory Medicine and Allergology, Kochi Medical School, Kochi University, Kochi, Japan; e Division of Respiratory Medicine, Department of Internal Medicine, Kobe University Graduate School of Medicine, Kobe, Japan; f Department of Respiratory Medicine, Nagoya University, Nagoya, Japan; g Department of Respiratory Medicine, Faculty of Life Sciences, Kumamoto University, Kumamoto, Japan; h Division of Pulmonary Medicine, Department of Medicine, Keio University School of Medicine, Tokyo, Japan; i Department of Pulmonary Medicine, Faculty of Medicine, University of Tsukuba, Tsukuba, Japan; j Division of Respiratory Medicine and Rheumatology, Department of Multidisciplinary Internal Medicine, School of Medicine, Faculty of Medicine, Tottori University, Tottori, Japan; k Department of Respiratory Medicine and Allergology, Department of Medicine, Teikyo University School of Medicine, Tokyo, Japan; l Department of Molecular and Internal Medicine, Graduate School of Biomedical and Health Sciences, Hiroshima University, Hiroshima, Japan; m Department of Respiratory Medicine, Tokyo Women’s Medical University, Tokyo, Japan; n Department of Respiratory Medicine, Juntendo University Faculty of Medicine and Graduate School of Medicine, Tokyo, Japan; o Department of Respiratory Medicine, Tohoku University Graduate School of Medicine, Sendai, Japan; p Department of Respiratory Medicine, NHO Toneyama Medical Center, Osaka, Japan; q Department of Respiratory Medicine and Allergy, Tosei General Hospital, Aichi, Japan; r Department of Respiratory Medicine, Hamamatsu Rosai Hospital, Hamamatsu, Japan; s Department of Respiratory Medicine, Osaka Habikino Medical Center, Osaka, Japan; t Division of Pulmonary Medicine, Department of Internal Medicine, Tachikawa Hospital, Tokyo, Japan; u Department of Respiratory Medicine, NHO Kanazawa Medical Center, Kanazawa, Japan; v Department of Respiratory Medicine, Kobe City Medical Center West Hospital, Kobe, Japan; w Department of Respiratory Medicine, Kobe City Medical Center General Hospital, Kobe, Japan; x Department of Respiratory Medicine, Akashi Medical Center, Hyogo, Japan; y Department of Respiratory Medicine, NHO Fukuokahigashi Medical Center, Fukuoka, Japan; z Department of Respiratory Medicine, Iizuka Hospital, Fukuoka, Japan; aa Department of Respiratory Medicine, Iwata City Hospital, Shizuoka, Japan; bb Department of Respiratory Medicine, Fujita Health University, Toyoake, Aichi, Japan; cc Department of Internal Medicine, Tokushima Prefecture Naruto Hospital, Tokushima, Japan; dd Department of Respiratory Medicine and Allergology, Kindai University Nara Hospital, Ikoma, Japan

**Keywords:** *Aspergillus* sensitization, ABPA, bronchiectasis, mucus plug, monocyte, refractory asthma

## Abstract

**Background:**

Sensitization to *Aspergillus,* mucus plugs, and bacterial colonization may coexist and relate to a refractory phenotype during follow-up in asthma with bronchiectasis and allergic bronchopulmonary aspergillosis (ABPA).

**Objective:**

This study aimed to clarify the features of *Aspergillus*-sensitized refractory asthma with bronchiectasis and determine the refractory phenotype in this population and ABPA.

**Methods:**

This study included cases of the oldest available *Aspergillus fumigatus*–specific IgE data and chest computed tomography images from a nationwide survey of refractory asthma with bronchiectasis. The characteristics of the *A fumigatus*–IgE positive (*Af* sIgE^+^) group were investigated and compared with its nonsensitized counterpart (*Af* sIgE^−^) and ABPA group. Cluster analysis was conducted to determine the refractory phenotype.

**Results:**

The *Af* sIgE^+^ group (n = 35) demonstrated type 2 inflammation levels intermediate between the ABPA (n = 42) and *Af* sIgE^−^ (n = 38) groups while exhibiting higher blood monocyte counts than the *Af* sIgE^−^ group. Cluster analysis conducted in patients with ABPA and *Af* sIgE^+^ newly determined 2 clusters: one was characterized by a younger age of asthma onset with fungal detection in sputum, and the other was characterized by mucus plugs and inflammation with eosinophils and monocytes, which was significantly related to mucus plugs, airflow limitation, and trend to show exacerbation. In the latter cluster, mucus plugs persisted, and 30% yielded *Pseudomonas aeruginosa* in the sputum <5 years later.

**Conclusion:**

The refractory phenotype with persistent mucus plugs was identified in *Aspergillus*-sensitized refractory asthma with bronchiectasis and ABPA. Mucus plug prevention is warranted.

Allergic bronchopulmonary aspergillosis (ABPA) or mycosis (ABPM) is a classic type of central bronchiectasis associated with severe asthma. Sensitization to *Aspergillus fumigatus,* which enhances type 2 inflammation, is also observed in 11% to 24% of severe asthma without ABPA^1^, ^2^, ^3^ and is associated with poor asthma control, which has been termed severe asthma with fungal sensitization. Not only asthma but also 29.5% to 76.5% of patients with non–cystic fibrosis bronchiectasis are sensitized to *A fumigatus.*^4^^,^^5^ Therefore, a broader term for allergic fungal airway disease has been proposed to encompass ABPA and other conditions with IgE sensitization to thermotolerant fungi and evidence of fungus-related lung damage,^6^^,^^7^ which highlights the importance of sensitization to thermotolerant fungi in the pathophysiology across airway diseases.

We have recently reported that cases with refractory asthma with bronchiectasis are heterogeneous, ranging from type 2–high to type 2–low phenotypes.^8^ Refractory asthma with bronchiectasis may have a phenotype sensitized to *A fumigatus* that shares several features with ABPA while retaining features of bronchiectasis other than ABPA. In contrast, 50% of ABPA patients developed chronic lower respiratory tract infections during the clinical course, mostly with *Staphylococcus aureus,* followed by *Pseudomonas aeruginosa* and nontuberculous mycobacteria,^9^ although the development of lower airway infection in ABPA has been rarely reported.^10^ One may encounter cases in clinical settings in which differentiating between the two conditions is difficult (ie, *A fumigatus*–sensitized refractory asthma with bronchiectasis and long-standing ABPA). Identifying such cases may also be important alongside clear differentiation because affected patients may be the most likely to present with severe type 2–high immune responses and inflammation with bacterial colonization, which may cause a poor clinical outcome. However, no such phenotype has been determined.

Recently, a study highlighted the importance of mucus plugs and proposed the disease entity of “muco-obstructive lung disease,” which encompasses chronic obstructive pulmonary disease, cystic fibrosis, primary ciliary dyskinesia, and non–cystic fibrosis bronchiectasis.^11^ A recent study of bronchiectasis revealed that IL-1β–expressing macrophage is upregulated in bronchiolar mucus plugs,^12^ whereas mucus plugs in the central airways are filled with eosinophils and Charcot-Leyden crystals in ABPA.^13^ Mucus plugs are frequently associated with airflow limitation, causing ventilation defects,^14^ regardless of the cell types responsible for mucus plugs. Furthermore, mucus plugs can be a source of chronic infection because they cause local hypoxia,^15^ where *P aeruginosa* can survive^16^ and coexist with *S aureus,* resulting in worse clinical courses.^17^ Therefore, mucus plugs could be the key factor that determines the refractory phenotype.

First, this study of refractory asthma comorbid with bronchiectasis aimed to clarify the characteristics of *Aspergillus* sensitization in refractory asthma with bronchiectasis (*Af* sIgE^+^) compared with its nonsensitized counterpart (*Af* sIgE^−^) and ABPA. Second, this association study investigated the role of mucus plugs in *Af* sIgE^+^ and ABPA and conducted a cluster analysis to determine the refractory phenotype in *Af* sIgE^+^ and ABPA.

## Methods

### Study design and population

The bronchiectasis and asthma (BEXAS) study was a nationwide survey conducted at accredited and affiliated facilities of the Japanese Respiratory Society and Japanese Society of Allergology. This study included patients with refractory asthma complicated by bronchiectasis, bronchiolitis, or both, with persistent sputum symptoms, and with a history of visits from January 2015 to September 2019. This study defined refractory asthma as asthma that was refractory to standard treatment and management, irrespective of inhaled corticosteroid doses. The patient exclusion criteria and details of the questionnaire and laboratory data have been previously described elsewhere.^8^ Our analysis included patients with the oldest available *A fumigatus*–specific IgE (*Af* sIgE) data and chest computed tomography (CT) images. Additionally, cases of ABPA that met Rosemberg’s,^18^ Asano’s,^19^ or other criteria, including the International Society for Human and Animal Mycology’s,^20^ were included. The phase of ABPA (ie, stable or acute/recurrent phase when the data were obtained) was documented. The phase of *Af* sIgE^+^ group (ie, stable or exacerbated phase at data acquisition) was also documented for the data used in the cluster analysis. The oldest available *Af* sIgE of ≥0.35 UA/mL was considered positive for *A fumigatus* sensitization. The oldest data in the last 5 years were obtained for laboratory and pulmonary function data, and the earliest available data were requested for CT data. The types of exacerbation were categorized into 3 exacerbations requiring systemic corticosteroids, antibiotics, and admission in the last 2 years. Causes of exacerbations—asthma, bronchiectasis, or ABPA—were not determined. Among the comorbidities, neutrophilic sinusitis was a diagnosis of exclusion and was used if the sinusitis did not meet the criteria of eosinophilic chronic rhinosinusitis proposed in the Japanese Epidemiological Survey of Refractory Eosinophilic Chronic Rhinosinusitis Study: JESREC score.^21^ This study was approved by the Kyoto University medical ethics committee (R2168).

### Analysis of chest CT images

Bronchodilation was defined as an enlarged bronchoarterial ratio of ≥1.1 or lack of tapering of the airway toward the periphery.^8^ The degree of bronchodilatation was assessed by the modified Reiff score.^22^ Bronchiolitis was defined when centrilobular nodules or tree-in-bud signs were present in one or more lobes, and its severity was assessed by counting the number of affected lobes. The lingula was defined as 1 lobe, and a total of 6 lobes were evaluated. The mucus score was assessed as previously reported only for patients with available high-resolution CT images.^14^ Briefly, high-resolution CT images were confirmed from 3 directions, and the opacified area of the airway lumen, contiguous with the open airway lumen, was considered the mucus plug. The scoring system was based on bronchopulmonary segmental anatomy: a total of 10 (1-10) segments for the right lung, and segments 1 + 2, 3, 4, 5, 6, 8, 9, and 10 for the left lung. Each bronchopulmonary segment was assigned a score of 1 (mucus plug present) or 0 (mucus plug absent). The segment scores of each lobe were summed to generate a total mucus score for both lungs. The airway size where mucus plugs were present was not identified.

### Statistical analysis

JMP 16 software (SAS Institute) was used for analyses, except for the cluster analysis. The chi-square test, Fisher exact test, Wilcoxon rank-sum test, and Kruskal-Wallis test were used to compare 2 or more groups, where appropriate. The Steel-Dwass test was used for multiple comparison tests. The Cochran-Armitage test for trend was conducted for categorical data of blood eosinophil counts. Patients that were followed up for at least the corresponding period were included when analyzing the frequencies of episodes, such as exacerbations in a defined period. A *K*-prototype clustering was performed by R 4.4.1 software (R Project; www.r-project.org) with the following 6 variables: 4 from the diagnostic criteria for ABPM,^19^ comprising detection of *Aspergillus* spp by sputum culture, blood eosinophil count of ≥500/μL, serum total IgE of ≥417 IU/mL, and *Af* sIgE of ≥0.35 UA/mL; and 2 reflecting bronchiectasis and antifungal inflammation, comprising blood neutrophil and monocyte counts. Data on immunoprecipitation against *Aspergillus* or anti–*Aspergillus*-specific IgG were not available and thus are not included in this cluster analysis. The oldest or earliest available data for blood, spirometry, sputum, and radiologic findings, which we believed to be less influenced by treatment, were used for data analysis, including cluster analysis, unless otherwise stated. The number of responses for each variable is provided in the tables and figure captions. *P* < .05 was considered statistically significant. Data are presented as means (standard deviations [SDs]).

## Results

### Characteristics of *Af* sIgE^+^ refractory asthma with bronchiectasis

This study enrolled 115 patients from 41 centers, with a mean (SD) age of 65 (15) years and 61% female (n = 70), categorized into groups as follows: ABPA (n = 42), *Af* sIgE-positive without ABPA (*Af* sIgE^+^) (n = 35), and *Af* sIgE-negative (*Af* sIgE^−^) (n = 38). Therefore, *Af* sIgE positivity in asthma with bronchiectasis without ABPA was 48%. Of the 42 patients with ABPA, 31 were diagnosed by Asano’s criteria and 11 by Rosenberg’s criteria. The *Af* sIgE^+^ group demonstrated similar patterns of auscultation results and current medications, and had neutrophilic sinusitis and diffuse panbronchiolitis obliterans with a similar frequency as the *Af* sIgE^−^ group (see Table E1 in the Online Repository available at www.jaci-global.org). Previous laboratory data (Table I) indicated that the *Af* sIgE^+^ group exhibited intermediate levels of blood eosinophil count, serum total IgE, and *Af* sIgE titer and frequencies of allergic sensitization (except for *A fumigatus* sensitization) between the ABPA and *Af* sIgE^−^ groups. Blood monocyte counts in the *Af* sIgE^+^ and ABPA groups were higher than those in the *Af* sIgE^−^ group. The 3 groups demonstrated no significant differences in exhaled nitric oxide level and percentage predicted forced expiratory volume in 1 second (%FEV_1_). In the present blood data, which were obtained 4.8 (SD 4.3) years after the previous data, the significant difference in blood monocyte among the 3 groups disappeared (see Table E2 in the Online Repository). The average (SD) periods between the previous and present CT data and spirometry were 4.9 (4.3) years and 4.3 (4.1) years, respectively. In the ABPA group, 63.2% of the previous data were obtained during the acute phase or diagnostic timing, and 15.8% of the present data were obtained during the acute phase.

**Table I:** Patient characteristics and previous laboratory imaging results

Characteristic	ABPA		Non-ABPA				*P*
	ABPA	No.	*Af* sIgE^+^	No.	*Af* sIgE^−^	No.	
Female sex, no. (%)	24 (57.1)	42	18 (51.4)†	35	28 (73.7)	38	.12
Age (years)	62.2 (13.3)	39	68.0 (13.4)	35	65.9 (17.0)	38	.14
Body mass index (kg/m^2^)	21.7 (4.8)	25	21.4 (3.7)	29	22.7 (4.1)	29	.38
Smoking history (%)		42		33		37	.39
No	83		73		78		
Current	12		27		19		
Past	5		0		3		
Age (years) at diagnosis of asthma	36.3 (23.6)	38	35.3 (24.5)	31	45.2 (23.2)	37	.15
Period (years) from asthma diagnosis to airway lesion diagnosis	18.5 (20.8)	36	23.6 (24.1)	28	12.4 (18.0)	32	.31
Childhood pneumonia, no. (%)	2 (5.4)	37	2 (6.1)	33	7 (20.0)	35	.08
**Previous laboratory and image data**							
Blood test							
WBC (μL)	8252 (3017)	39	8511 (3226)	35	7532 (2959)	36	.30
Neutrophil (μL)	4610 (1946)	38	5812 (3387)	32	5165 (3247)	34	.51
Monocyte (μL)	453 (213)	38	439 (226)†	34	329 (137)∗	36	.006
Eos (μL)	1181 (1369)	40	606 (524)	33	409 (575)∗	34	.0002
Eos ≥300/μL (%)	78	40	70†	33	44∗	34	.051‡
Eos ≥500/μL (%)	70	40	48	33	26∗	34	.002‡
Basophil (μL)	60 (41)	36	52 (57)	33	37 (32)∗	33	.04
Total IgE (IU/mL)	3068 (4575)	40	1569 (3000)†	30	357 (521)∗	31	<.0001
*Aspergillus*-specific IgE (UA/mL)	18 (21)	42	10 (15)∗†	35	0.2 (0.1)∗	37	<.0001
Specific IgE against:							
Alternaria (+), no. (%)	19 (76.0)	25	10 (38.5)∗†	26	0∗	26	<.0001
Cat dander (+), no. (%)	14 (56.0)	25	3 (13.6)∗	22	1 (3.2)∗	31	<.0001
Dog dander (+), no. (%)	14 (58.3)	24	2 (10.0)∗	20	2 (7.7)∗	26	<.0001
Orchard grass (+), no. (%)	8 (32.0)	25	6 (26.1)	23	5 (20.8)	24	.67
Mugwort (+), no. (%)	11 (47.8)	23	4 (22.2)	18	4 (14.8)∗	27	.03
Cedar (+), no. (%)	18 (72.0)	25	17 (65.4)†	26	13 (38.2)∗	34	.02
House dust mite (+), no. (%)	20 (76.9)	26	16 (57.1)†	28	9 (26.5)∗	34	.0004
FEV_1_/FVC (%)	65.6 (12.8)	28	66.0 (13.4)	28	64.6 (11.8)	25	1.00
%FEV_1_ (%)	66.1 (27.7)	27	77.4 (22.4)	27	74.2 (25.3)	25	.23
Exhaled nitric oxide (ppb)	61.9 (55.9)	17	36.6 (23.4)	27	64.5 (63.0)	26	.25
Sputum culture, no. (%)							
*Aspergillus* spp (+)	7 (19.4)	36	2 (6.5)	31	0∗	33	.02
Fungus (+)	8 (22.2)	36	2 (6.5)	31	0∗	33	.007
*Pseudomonas aeruginosa* (+)	6 (17.1)	35	6 (20.0)	30	6 (19.4)	31	.95
Gram-negative bacteria (+)	10 (28.6)	35	15 (50.0)	30	15 (48.4)	31	.14
Airway lesions							
Modified Reiff score	3.8 (4.2)	41	2.5 (3.4)	32	2.9 (3.3)	35	.30
No. of bronchiolitis-affected lobes	3.1 (2.1)	40	2.9 (2.2)	31	2.8 (2.3)	34	.81
Mucus score	7.0 (4.8)	19	5.4 (6.0)	8	5.9 (3.8)	11	.47
Presence of exacerbations and bronchopneumonia in last 2 years, no. (%)							
Requiring systemic corticosteroids	18 (56.3)	28	12 (42.9)	28	10 (34.5)	29	.22
Requiring antibiotics	14 (45.2)	31	14 (48.3)	29	10 (33.3)	30	.47
Bronchopneumonia	12 (38.7)	31	12 (41.4)	29	9 (30.0)	30	.64

Data are shown as means (SDs) unless otherwise noted. *P* value demonstrates variance across 3 groups. *(+)* indicates positive result.

*Eos,* Eosinophils; *FVC,* forced vital capacity.

∗ *P* < .05 vs ABPA.

† *P* < .05 vs *Af* sIgE^−^ group.

‡ *P* < .05 by Cochran-Armitage test for trend.

### Association between mucus score and blood inflammatory cells or other features

**Fig 1:**
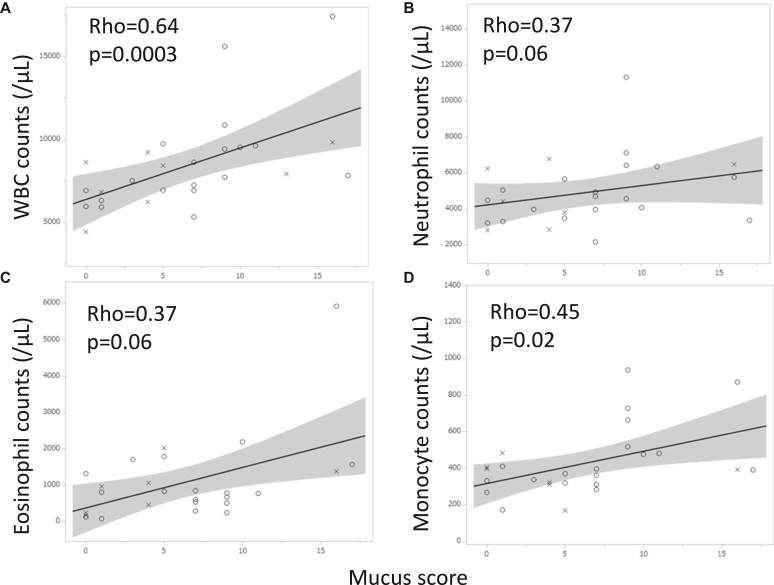
Associations between previous mucus score and WBC **(A)** as well as neutrophil **(B)**, eosinophil **(C)**, and monocyte **(D)** counts. *Circle* indicates ABPA; *cross, Af* sIgE^+^.

**Fig 2:**
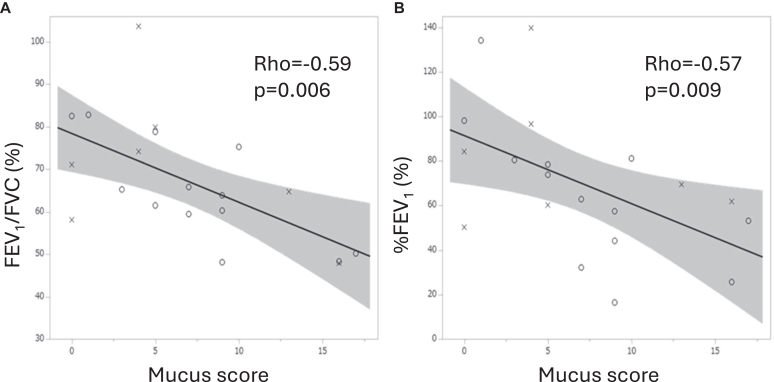
Associations between previous mucus score and FEV_1_/FVC **(A)** and %FEV_1_**(B)**. N = 20. *Circle* indicates ABPA; *cross, Af* sIgE^+^. *FVC,* Forced vital capacity.

**Fig 3:**
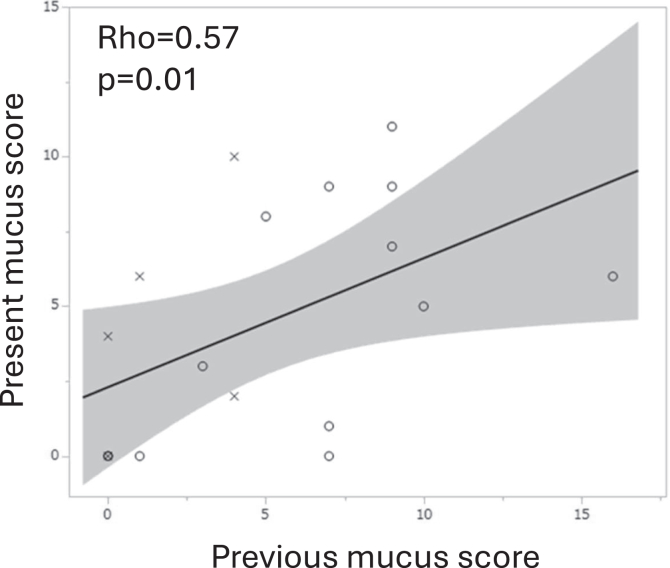
Relationship between previous and present mucus scores. *Circle* indicates ABPA; *cross, Af* sIgE^+^.

**Fig 4:**
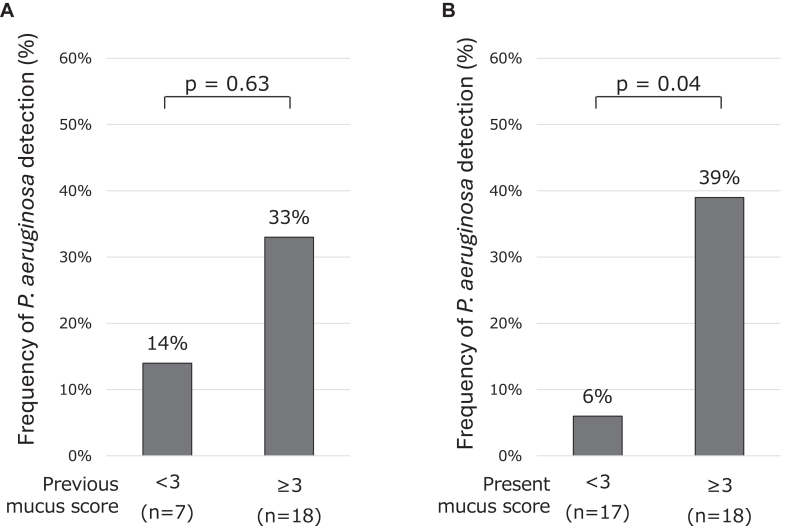
Frequencies of *Pseudomonas aeruginosa* detection in sputum of patients with ABPA or *Af* sIgE^+^ with mucus plugs (mucus score ≥3) compared with those with low mucus score (mucus score <3). Based on sputum obtained at time corresponding to previous **(A)** and present **(B)** mucus scores.

The roles of mucus plugging were then investigated in *Af* sIgE^+^ and ABPA. In the previous data, mucus score (n = 27) exhibited positive correlations with white blood cell count (WBC) (ρ = 0. 64, *P* = .0003) and monocytes (ρ = 0.45, *P* = .02), insignificant correlations with neutrophils (ρ = 0.37, *P* = .06) and eosinophils (ρ = 0.37, *P* = .06) (Fig 1), and significantly negative correlations with FEV_1_/forced vital capacity (n = 20, ρ = −0.59, *P* = .006) and %FEV_1_ (ρ = −0.57, *P* = .009) (Fig 2). These were true when the *Af* sIgE^−^ group was included in the analysis (n = 38) (see Table E3 in the Online Repository available at www.jaci-global.org). Previous and present mucus scores were significantly correlated (ρ = 0.57, *P* = .01) (Fig 3). The present mucus score (n = 41) was significantly associated with WBC (ρ = 0.44, *P* = .004), blood eosinophil count (ρ = 0.37, *P* = .02), monocyte count (ρ = 0.33, *P* = .04) (see Fig E1 in the Online Repository), and exacerbations requiring admissions (n = 36, ρ = 0.45, *P* = .006); and were insignificantly associated with exacerbations requiring systemic corticosteroids (ρ = 0.29, *P* = .08) in the last 2 years and insignificantly associated with neutrophil counts (ρ = 0.30, *P* = .06) (Fig E1) and %FEV_1_ (ρ = −0.34, *P* = .06) (see Fig E2 in the Online Repository). At present, the ratio of sputum detection of *P aeruginosa* was 39% in the higher mucus score group (≥3, median value), which was significantly greater than that in the lower mucus score (<3) group (Fig 4). This difference was not observed for the sputum detection of *Aspergillus* (data not shown).

### Cluster analysis

**Fig 5:**
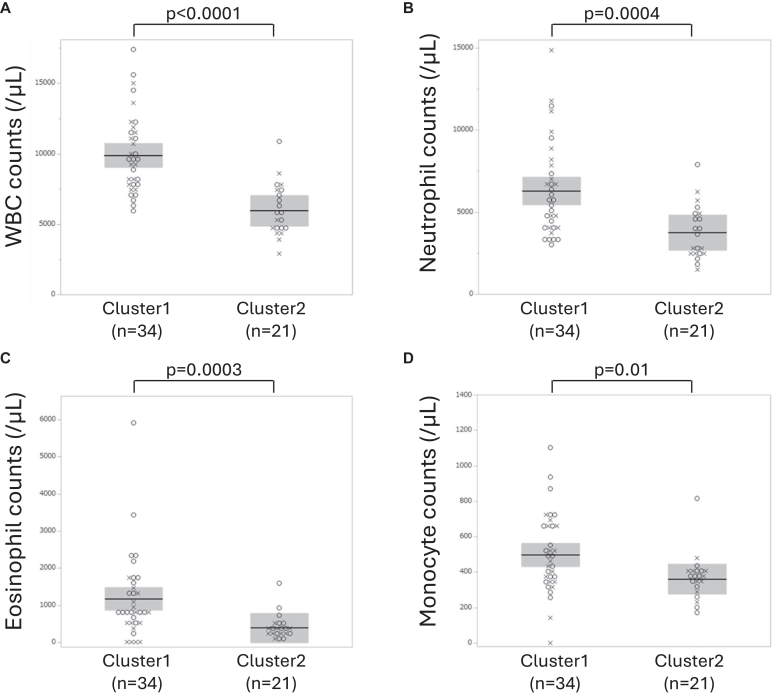
Previous blood data of WBC **(A)**, neutrophil **(B)**, eosinophil **(C)**, and monocyte **(D)** counts in each cluster. Box-and-whisker plots indicate medians and quartiles. *Circle* indicates ABPA; *cross, Af* sIgE^+^.

**Fig 6:**
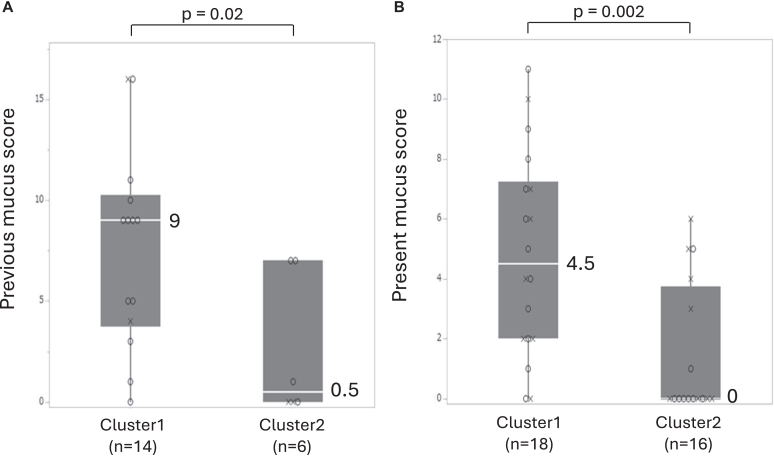
Mucus scores on previous **(A)** and present **(B)** chest CT images in each cluster. Box-and-whisker plots indicate medians and quartiles. *Circle* indicates ABPA; *cross, Af* sIgE^+^.

A cluster analysis was conducted in the combined population of ABPA and *Af* sIgE^+^ to identify the refractory phenotype in patients sensitized to *Aspergillus.* The cluster analysis using 4 variables from the diagnostic criteria for ABPM and previous blood monocytes and neutrophils determined 2 clusters. Clusters 1 (n = 34) and 2 (n = 21) similarly included patients from the ABPA and *Af* sIgE^+^ groups and had comparable serum total IgE and *Af* sIgE levels. Overall, 48% of the data input in the analysis were obtained at the stable phase and 52% at the acute, recurrent, or exacerbated phases. Cluster 1, 56% of which were from the ABPA group and 44% from the *Af* sIgE^+^ group, demonstrated higher WBC, eosinophil and monocyte counts (Fig 5), and mucus scores (Fig 6) than cluster 2 both at previous (Table II) and present data (see Table E4 in the Online Repository available at www.jaci-global.org). Additionally, cluster 1 exhibited significantly higher blood neutrophil counts in previous data and currently lower %FEV_1_, and appeared to have more frequent exacerbations requiring systemic corticosteroids in the last 2 years (*P* = .07) and *P aeruginosa* detection (30.0% vs 5.6%, *P* = .07) in present sputum than cluster 2. Meanwhile, cluster 2, 57% of which were from the ABPA group and 43% from the *Af* sIgE^+^ group, exhibited higher detection rate of fungi (*P* = .04) and *Aspergillus* spp (*P* = .09) in previous sputum cultures than cluster 1 (Table Ⅱ). Further, the age at onset of asthma was significantly younger, and the period from diagnosis of asthma to airway lesions was significantly longer in cluster 2 than in cluster 1. Data obtained at the acute phase tended to be more frequently included in cluster 1 than in cluster 2 (62% vs 33%, *P* = .08) for previous data, but were comparable for present data (15% vs 22%, *P* = .70).

**Table II:** Patient characteristics, and previous laboratory and imaging data by cluster

Characteristic	Cluster				*P*
	Cluster 1	No.	Cluster 2	No.	
Female sex, no. (%)	12 (57.1)	34	15 (44.1)	21	.35
Age (years)	64.3 (14.7)	33	68.0 (12.8)	21	.37
Body mass index (kg/m^2^)	21.3 (4.9)	23	22.1 (3.4)	19	.36
Smoking history (%)		33		21	.46
No	72		86		
Current	24		14		
Past	3		0		
Age (years) at diagnosis of asthma	40.3 (23.1)	29	26.1 (23.3)	20	.03
Period (years) from asthma diagnosis to airway lesion diagnosis	12.1 (14.7)	24	34.2 (27.0)	20	.007
Childhood pneumonia, no. (%)	0	31	2 (10.0)	20	.15
**Previous laboratory and image data**					
Blood test					
WBC (μL)	9899 (2885)	34	5960 (1845)	21	<.0001
Neutrophil (μL)	6298 (3896)	34	3758 (1671)	21	.0004
Monocyte (μL)	497 (228)	34	361 (130)	21	.01
Eos (μL)	1176 (1139)	34	390 (346)	21	.0003
Eos ≥300/μL (%)	85	34	52	21	.008
Eos ≥500/μL (%)	82	34	19	21	<.0001
Basophil (μL)	63 (51)	33	41 (25)	21	.20
Total IgE (IU/mL)	3167 (5325)	34	1756 (1874)	21	.87
*Aspergillus*-specific IgE, UA/mL	15.1 (21.9)	34	17.8 (19.8)	21	.51
Specific IgE against:					
Alternaria (+), no. (%)	15 (68.2)	22	7 (50.0)	14	.31
Cat dander (+), no. (%)	10 (47.6)	21	4 (28.6)	14	.31
Dog dander (+), no. (%)	10 (50.0)	20	4 (40.0)	10	.71
Orchard grass (+), no. (%)	5 (26.3)	19	6 (42.9)	14	.46
Mugwort (+), no. (%)	9 (52.9)	17	4 (33.3)	12	.45
Cedar (+), no. (%)	14 (63.6)	22	12 (85.7)	14	.25
House dust mite (+), no. (%)	15 (62.5)	24	12 (85.7)	14	.16
FEV_1_/FVC (%)	66.7 (13.2)	26	68.8 (12.6)	15	.75
%FEV_1_ (%)	67.3 (25.5)	25	82.5 (24.6)	15	.09
Exhaled nitric oxide (ppb)	42.3 (35.0)	21	51.3 (58.3)	13	.94
Sputum culture, no. (%)					
*Aspergillus* spp (+)	2 (5.9)	34	5 (23.8)	21	.09
Fungus (+)	2 (5.9)	34	6 (28.6)	21	.04
*Pseudomonas aeruginosa* (+)	7 (21.2)	33	4 (19.1)	21	1.00
Gram-negative bacteria (+)	12 (36.4)	33	8 (38.1)	21	.90
Airway lesions					
Modified Reiff score	3.4 (4.4)	32	2.7 (3.5)	20	.79
No. of bronchiolitis-affected lobes	3.2 (2.1)	32	2.7 (2.4)	20	.46
Mucus score	7.6 (4.9)	14	2.5 (3.5)	6	.02
Presence of exacerbations and bronchopneumonia in last 2 years, no. (%)					
Requiring systemic corticosteroids	18 (66.7)	27	8 (40.0)	20	.07
Requiring antibiotics	12 (46.2)	26	10 (50.0)	20	.80
Bronchopneumonia	10 (38.5)	26	8 (40.0)	20	.92

Data are shown as means (SDs) unless otherwise noted. *(+)* indicates positive result.

*Eos,* Eosinophils; *FVC,* forced vital capacity.

## Discussion

To our knowledge, ours is the first study to confirm the characteristics of *A fumigatus*–sensitized refractory asthma with bronchiectasis and determine the refractory phenotype in refractory asthma with bronchiectasis and ABPA using cluster analysis. Overall, *Af* sIgE positivity was 48% in refractory asthma with bronchiectasis without ABPA, and the *Af* sIgE^+^ group demonstrated type 2 inflammation levels intermediate between the ABPA and *Af* sIgE^−^ groups while exhibiting higher monocyte counts than *Af* sIgE^−^ group. In the combined population of the *Af* sIgE^+^ and ABPA groups, the mucus score was associated with blood inflammatory cell counts, airflow limitation, exacerbations, and, later, with detection of *P aeruginosa* in sputum. Finally, cluster analysis determined a refractory cluster, consisting of both the *Af* sIgE^+^ group and ABPA, which was characterized by elevated blood eosinophil and monocyte counts, mucus plugs, and poor clinical outcomes.

This study revealed that the rate of *A fumigatus* sensitization was 48%, which is comparable to previous results of severe asthma with bronchiectasis other than ABPA, rating 52.9%,^2^ and numerically higher than those with severe asthma alone, rating 11% to 24%.^1^^,^^2^ The increased sensitization rate in cases of comorbid bronchiectasis is considered to be due to the retention of *Aspergillus* conidia in the damaged airways with impaired mucociliary clearance and subsequent hyphae germination and formation, which are antigenic and trigger T_H_2 inflammation.^23^ Expectedly, the *Af* sIgE^+^ refractory asthma with bronchiectasis shared, although to a lesser degree, characteristics of enhanced type 2 inflammation, such as elevated blood eosinophil count and serum total IgE level with ABPA, while sharing several clinical features, including auscultation results and comorbidities with the *Af* sIgE^−^ group. Furthermore, the *Af* sIgE^+^ group as well as the ABPA group demonstrated higher monocyte counts than did the *Af* sIgE^−^ group. ABPA underdiagnosis in the *Af* sIgE^+^ group is a risk, but these results indicate that inflammation with eosinophils and monocytes may be crucial in allergic fungal airway disease.

Mucus plugs play a crucial role in the pathophysiology of various airway diseases. The combined population of *Af* sIgE^+^ and ABPA indicated that the mucus score was negatively correlated with %FEV_1_ and positively correlated with the frequency of exacerbations requiring admissions in the last 2 years, which was consistent with previous studies of asthma.^14^^,^^24^ Additionally, a correlation was found between previous and present mucus scores, confirming that mucus plugs are likely to persist.^24^ The mucus score was associated with blood eosinophil and monocyte counts and less strongly with blood neutrophil counts, both in the previous and present data. Additionally, *P aeruginosa* was more frequently detected in the sputum in the presence of mucus plugs. These data extend the previous results that revealed associations between mucus plugs and eosinophils in ABPA^25^ and in moderate to severe asthma that included bronchiectasis in 18%^26^ by exhibiting associations between mucus plugs and inflammation with monocytes, eosinophils, and neutrophils. These excessive responses and bacterial colonization, particularly by *P aeruginosa,* may attenuate the sensitivity to corticosteroid treatment and complicate the clinical course of patients with ABPA and *Af* sIgE^+^ with mucus plugs. Low-dose macrolide therapy was introduced in patients with bacterial colonization, but its immune-modulatory and anti-inflammatory effects may have been insufficient in the presence of mucus plugs. Meanwhile, the results should be carefully interpreted because they may be different from data from all newly diagnosed patients.

Cluster analysis was performed to identify the refractory phenotype. The mucus score was excluded from the cluster analysis because of the small number of subjects evaluated for the mucus score. Instead, blood neutrophil and monocyte counts were input as variables on the bases of the crucial roles of blood neutrophil count in bronchiectasis^27^^,^^28^ and monocytes or monocyte-derived macrophages in *Aspergillus*-related lung diseases,^29^^,^^30^ and mucus production in bronchiectasis^12^ and the association analysis of this study. Cluster 2 was characterized by a younger onset of asthma with higher fungal detection rates. Cluster 1 was characterized by mucus plugs, airflow limitation, and poor clinical outcomes. Furthermore, cluster 1 tended to later cause *P aeruginosa* colonization in the airways. This development may be associated with the persistence of mucus plugs because mucus plugs cause local hypoxia, where *P aeruginosa* survived^11^^,^^16^ in the absence of oxygen by being supplied with energy^31^ and adapting to the environment.^32^ The mechanisms underlying the difference between clusters 1 and 2 remain unknown. Oral corticosteroid and antifungal drug administration did not differ between the 2 clusters in this study. Older age at asthma onset and shorter time between asthma and airway lesion diagnosis, as observed in cluster 1, may contribute to developing a refractory phenotype. Regardless of the mechanisms, the differences in mucus scores and WBC as well as eosinophil and monocyte counts between the 2 clusters remained significant in both previous and present data, regardless of the timing of data acquisition, indicating the importance of the presence of mucus plugs. Mucus plug eradication and prevention are essential for better management.

The main player in ABPA and related diseases is eosinophils, and the role of monocytes or monocyte-derived macrophages is limited. Indeed, blood eosinophil counts were much higher in cluster 1 than in cluster 2. However, blood monocyte counts in cluster 1 were also elevated in both previous and present data. Along with eosinophils, macrophages or monocyte-derived macrophages may play a role in *Aspergillus*-sensitized bronchiectasis and ABPA as an initial key player in muco-obstructive lung disease^11^^,^^12^ and as a commander in the detection and defense against *Aspergillus*.^29^^,^^30^ Systemic monocytes may be recruited by *Aspergillus* conidia retention in the damaged airways, but the monocyte-derived macrophages may have attenuated the killing response of conidia in the type 2 inflammatory milieu, which may cause the persistence of conidia, maintenance of type 2 and type 17 inflammation, and mucus hypersecretion.^33^ A study of bronchiectasis revealed that patients with fungal sensitization were characterized by an increasing trend in sputum positivity of galactomannan and elevated sputum levels of the proinflammatory cytokines TNF-α and IL-1β, which are major cytokines produced by monocytes or monocyte-derived macrophages,^12^^,^^34^ which upregulate adhesion molecules in the endothelium and result in neutrophil and eosinophil airway recruitment.^35^

This study has some limitations. First, because of the retrospective nature of this study, the number of patients who met the eligibility criteria for this analysis was small, and the sampling timings of CT images and other examinations were inconsistent. However, the results of previous and present data in association and cluster analyses were consistent in many aspects, ensuring our certainty of the results. Second, we discussed only blood inflammatory cells; no data on airway inflammatory cells were presented. Third, the causes of exacerbations—be they exacerbations of asthma, bronchiectasis, or ABPA—were not determined, although exacerbation frequencies were associated with mucus score. Future prospective studies are required.

In conclusion, 48% of patients with refractory asthma with bronchiectasis were sensitized to *A fumigatus. Aspergillus*-sensitized refractory asthma with bronchiectasis and long-standing ABPA includes a refractory phenotype with mucus plugs and inflammation with eosinophils and monocytes, which is related to poor clinical outcomes. Mucus plug formation eradication and prevention are warranted.

## Disclosure statement

Supported by the Scientific Assembly of Allergy, Immunology & Inflammation, 10.13039/501100009098Japanese Respiratory Society, 10.13039/100030831Novartis Japan, and the 10.13039/100009619Japan Agency for Medical Research and Development (research grant 24ek0410097 for Allergic Disease and Immunology).

Disclosure of potential conflict of interest: K. Asano received lecturer fees from Sanofi, AstraZeneca, and Boehringer Ingelheim outside this work; and received a research grant on Allergic Disease and Immunology from the Japan Agency for Medical Research and Development. K. Fukunaga received lecturer fees from Sanofi, AstraZeneca, GlaxoSmithKline, Kyorin Pharmaceutical, Boehringer Ingelheim, and Novartis Pharma outside this work; and received grants from Boehringer Ingelheim and Chugai Pharmaceutical outside this work. N. Harada received lecturer fees from Sanofi, AstraZeneca, GlaxoSmithKline, Kyorin Pharmaceutical, and Novartis Pharma outside this work; and royalties from Sanofi, AstraZeneca, Daikin Investment, and TOSOH. T. Hirai received lecturer fees from AstraZeneca, Kyorin Pharmaceutical, and Boehringer Ingelheim outside this work. N. Hattori received lecturer fees from Sanofi, AstraZeneca, GlaxoSmithKline, Kyorin Pharmaceutical, Ono Pharmaceutical, MSD, and Pfizer Japan outside this work. T. Kimura received lecture fees from Sanofi, AstraZeneca, GlaxoSmithKline, Eli Lilly Japan, Chugai Phamaceutical, Novartis Pharma, Brsitol Myers Squibb, Meiji Seika Pharma, DAIICHI SANKYO, and MSD outside this work. H. Matsumoto received lecturer fees from Sanofi, AstraZeneca, GlaxoSmithKline, Kyorin Pharmaceutical, and Boehringer Ingelheim; received grants from Kyorin Pharmaceutical, Boehringer Ingelheim, and Teijin Pharma outside this work; and received support from the Japanese Respiratory Society and a research grant from Novartis Japan. O. Matsuno received lecturer fees from Sanofi, AstraZeneca, and GlaxoSmithKline. T. Sakagami received lecturer fees from AstraZeneca, GlaxoSmithKline, Novartis Pharma, and Boehringer Ingelheim outside this work. H. Sugiura received lecturer fees from Sanofi, AstraZeneca, GlaxoSmithKline, Novartis Pharma, and Boehringer Ingelheim outside this work. H. Sunadome reports grants from Philips Japan, ResMed, Fukuda Denshi, and Fukuda Lifetec Keiji outside this work. N. Tanabe received research grants from Daiichi Sankyo and FUJIFILM outside this work. K. Tomii received lecturer fees from Sanofi, AstraZeneca, GlaxoSmithKline, and Novartis Pharma outside this work. A. Yokoyama received lecturer fees from Sanofi, AstraZeneca, GlaxoSmithKline, and Boehringer Ingelheim outside this work. The rest of the authors declare that they have no relevant conflicts of interest.

## Supplementary data

Supplementary Figure E1

Supplementary Figure E2
