# Supplementary figures and images for: Refractory phenotype of Aspergillus-sensitized asthma with bronchiectasis and allergic bronchopulmonary aspergillosis
Source: J Allergy Clin Immunol Glob. 2024 Nov 1;4(1):100364. doi: 10.1016/j.jacig.2024.100364 (PMC11629325; doi:10.1016/j.jacig.2024.100364)

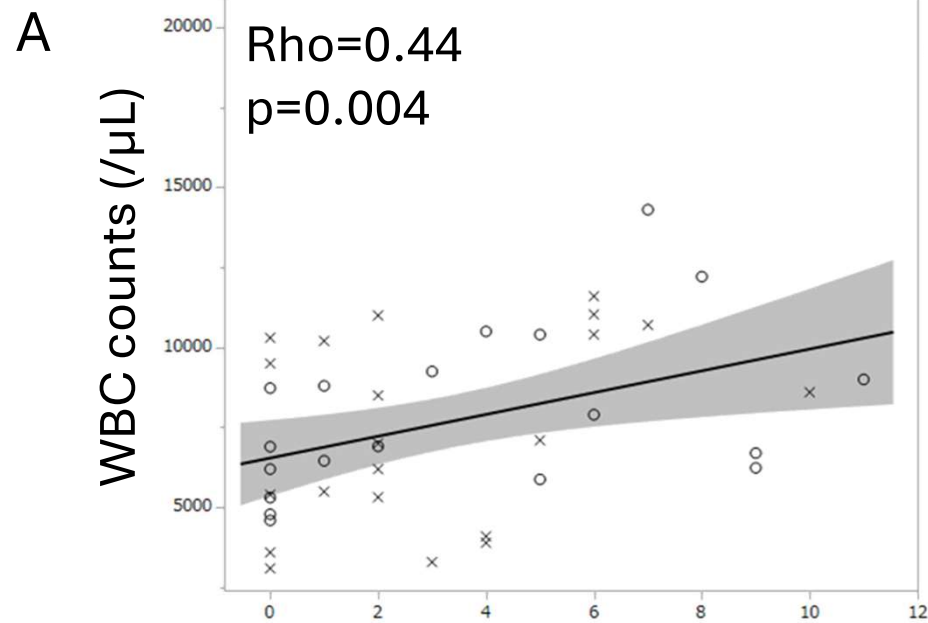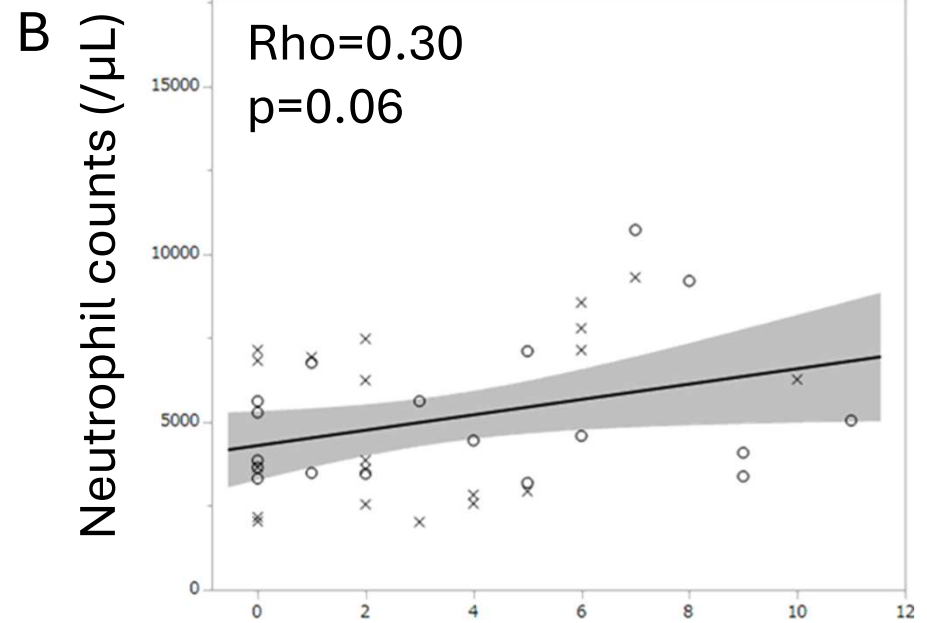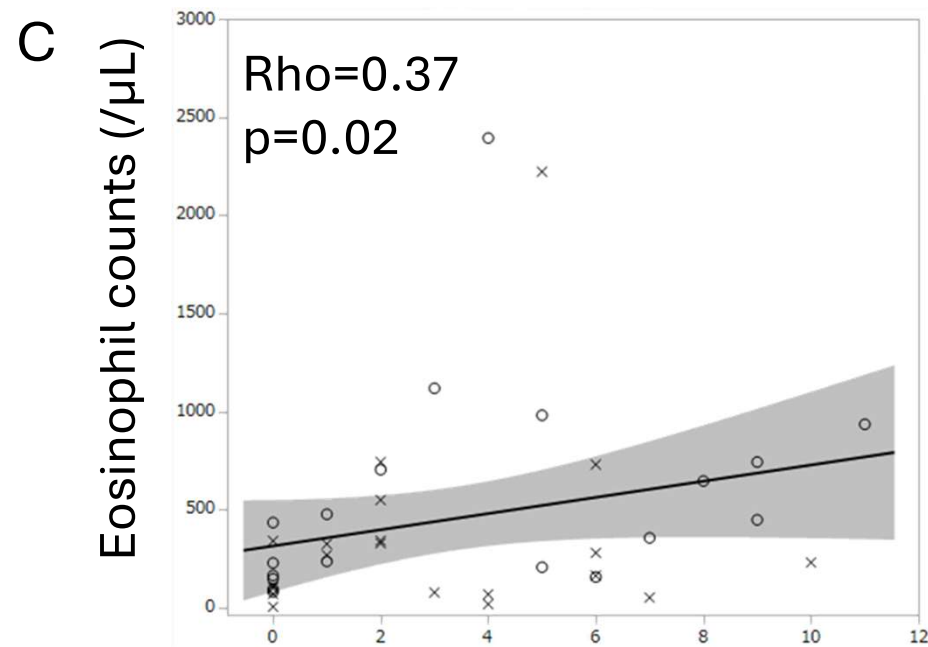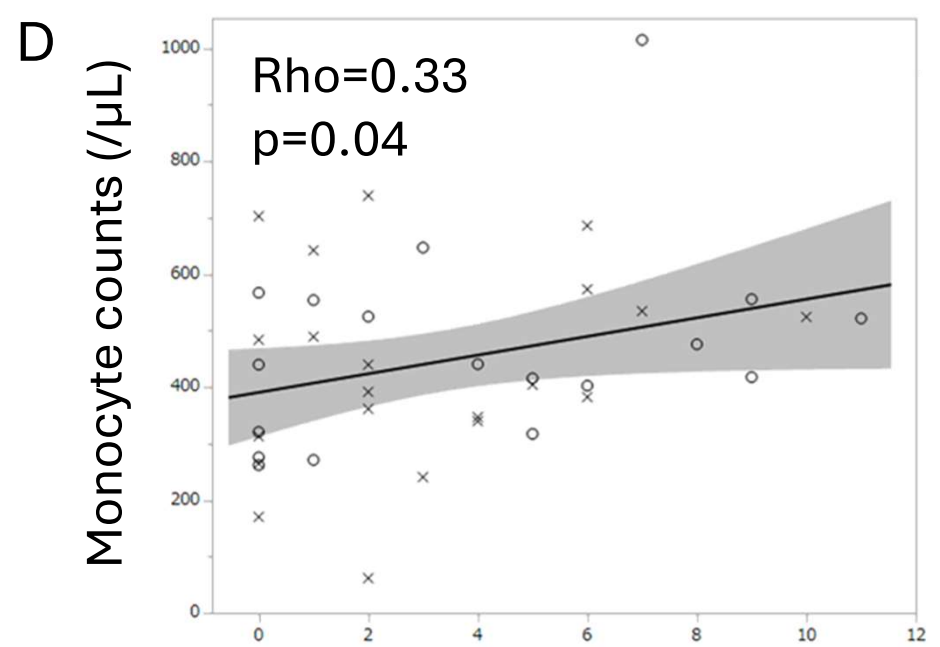

Present mucus score

Supplement: Supplementary Figure E1 [file mmc1.pdf]

A

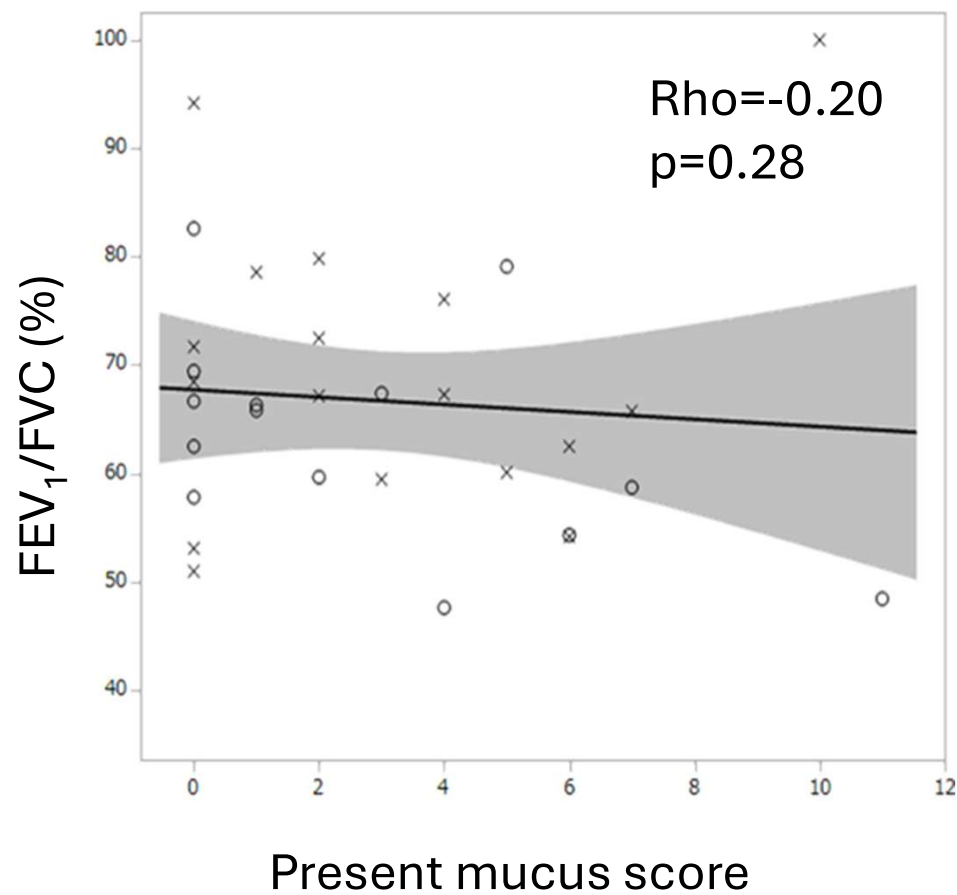

B

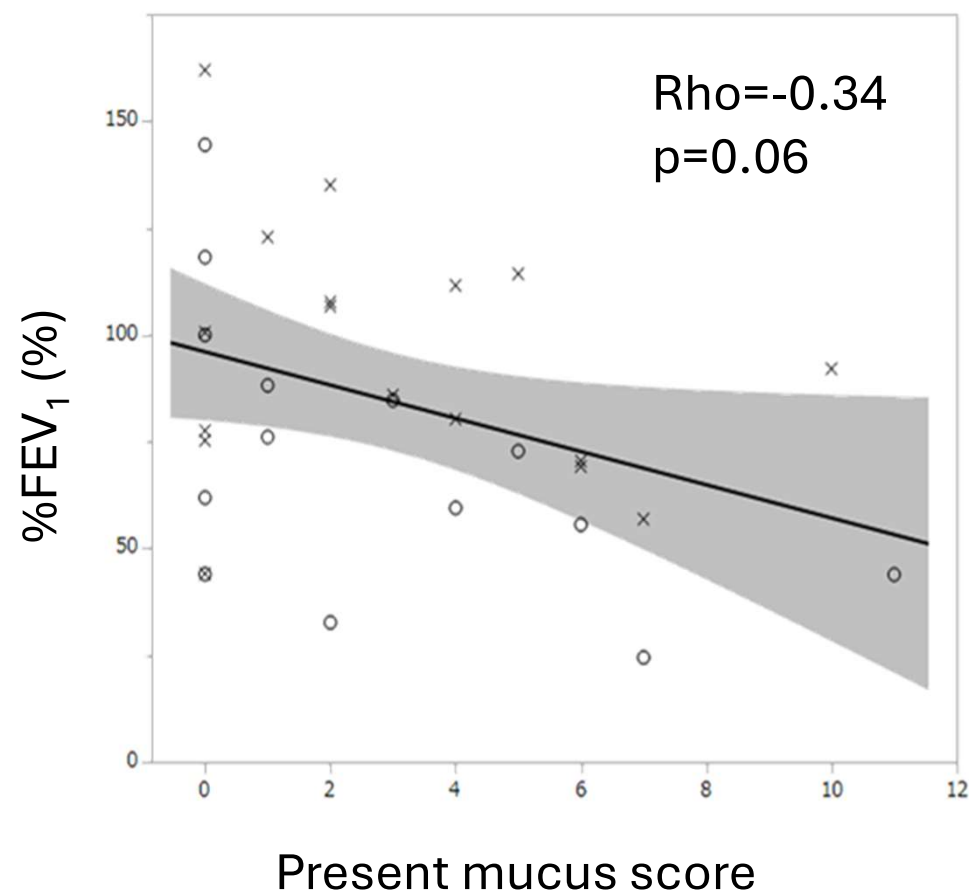

Supplement: Supplementary Figure E2 [file mmc2.pdf]
