# Supplementary material for: Refractory phenotype of Aspergillus-sensitized asthma with bronchiectasis and allergic bronchopulmonary aspergillosis
Source: J Allergy Clin Immunol Glob. 2024 Nov 1;4(1):100364. doi: 10.1016/j.jacig.2024.100364 (PMC11629325; doi:10.1016/j.jacig.2024.100364)
Supplement: Supplementary data [file mmc3.pdf]

Clean Online Repository

Refractory phenotype of *Aspergillus*-sensitized asthma with bronchiectasis and allergic bronchopulmonary aspergillosis

Figure legends

FIGURE E1. Relationships between present mucus scores and white blood cell (WBC) (A), neutrophil (B), eosinophil (C), and monocyte (D) counts. The circle indicates ABPA; the cross denotes *Af* sIgE$^+$.

FIGURE E2. Relationships between present mucus scores and FEV_1/FVC (A), and $\%FEV_1$ (B). The circle indicates ABPA; the cross denotes *Af* sIgE$^+$.

TABLE E1. Comorbidities, symptoms, auscultation findings, and treatments

	ABPA	N	Non-ABPA				P
			Af **sIgE⁺**	N	***Af*** **sIgE⁻**	N	
Comorbidities							
Chronic obstructive pulmonary disease, n (%)	1(2.6)	39	3(9.1)	33	1(2.9)	34	0.36
Diffuse panbronchiolitis, n (%)	0	37	7(21.2)*	33	5(15.2)*	33	0.02
Neutrophilic sinusitis, n (%)	6(15.4)	39	12(36.4)*	33	11(33.3)	33	0.09
Eosinophilic sinusitis, n (%)	3(7.9)	38	1(3.0)	33	7(20.6)	34	0.052
Pollinosis, n (%)	2(5.4)	37	5(15.2)	33	2(6.1)	33	0.28
Rheumatoid arthritis, n(%)	2(5.1)	39	3(9.1)	33	0	33	0.22
Diabetes mellitus, n (%)	2(5.1)	39	3(9.1)	33	4(11.8)	34	0.59
Gastroesophageal reflux disease, n (%)	0	37	3(9.1)	33	1(3.0)	33	0.14
Symptoms and auscultation findings							
Stable phase							
Cough, n (%)	10(25.0)	40	19(59.4)*	32	19(54.3)*	35	0.006
Rhonchi/wheeze, n (%)	3(7.9)	38	7(21.2)	33	8(25.0)	32	0.14
Coarse crackles, n (%)	1(2.6)	39	3(9.1)	33	5(15.6)	32	0.15
Exacerbation phase							
Cough, n (%)	33(82.5)	40	31(91.2)	34	30(81.1)	37	0.44
Rhonchi/wheeze, n (%)	20(54.1)	37	25(73.5)	34	29(82.9)*	35	0.02
Coarse crackles, n (%)	3(8.1)	37	8(23.5)	34	16(45.7)*	35	0.001
Treatments**							
Inhaled CS use (current), n (%)	38(90.5)	42	32(94.1)	34	33(91.7)	36	0.84
Inhaled CS dose (current), eq. fluticasone, μg/day	607(337)	41	763(423)	32	585(361)	36	0.11
Inhaled CS use (previous), n (%)	22(53.7)	41	26(81.3)*	32	19(61.3)	31	0.046
Inhaled CS dose (previous), eq. fluticasone, μg/day	319(367)	41	598(473)*	30	484(465)	31	0.04
Oral CS use, n (%)	32(78.1)	41	14(41.2)*	34	12(33.3)*	36	0.0001
Oral CS≥5mg/day, n (%)	26(65.0)	40	7(22.6)*	31	9(25.0)*	36	0.0001
Oral CS≥10mg/day, n (%)	17(42.5)	40	3(9.7)*	31	2(5.6)*	36	<.0001
LABA use, n (%)	33(82.5)	40	30(88.2)	34	36(97.3)	37	0.11
LAMA use, n (%)	10(25.0)	40	20(58.8)*	34	21(56.8)*	37	0.004
Macrolide therapy, n (%)	15(37.5)	40	20(58.8)	34	24(64.9)*	37	0.04
Antifungal agent, n (%)	17(40.5)	42	1(2.9)*	34	2(5.4)*	37	<.0001
Biologics use, n (%)	7(16.7)	42	13(38.2)*	34	9(24.3)	37	0.10
Airway clearance, n (%)	8(19.1)	39	14(41.2)	34	20(54.1)*	37	0.01

Data are shown mean (SD). P value in the last column demonstrates variance across the 3 groups. *p<0.05 vs ABPA.** Previously or currently used medications are presented, if not otherwise specified. CS, corticosteroids; LABA, long acting β_2 agonist; LAMA, long acting anti-muscarinic antagonist.

TABLE E2. Present data of the ABPA, *Af* sIgE^{+}, and *Af* sIgE^{-} groups

		ABPA	N	Non-ABPA				P
				***Af* sIgE^{+}**	**N**	***Af* sIgE^{-}**	**N**	
Blood test	White blood cell, /μL	7949(2860)	38	7854(3275)	35	7248(2466)	36	0.43
	Neutrophil, /μL	5188(2586)	38	5297(2945)	34	4920(2555)	34	0.70
	Monocyte, /μL	447(177)	38	460(164)	35	416(246)	36	0.16
	Eosinophil, /μL	482(447)	38	473(611)	35	325(408)*	35	0.04
	Basophil, /μL	59(70)	38	60(63)	35	36(32)	34	0.047
	Total IgE, IU/mL	1890(2732)	34	813(840)†	27	283(566)*	25	<.0001
FEV_1/Forced vital capacity, %		68.0(13.3)	27	64.9(14.0)	30	67.9(12.6)	30	0.51
%FEV_1, %		76.8(28.3)	27	84.3(31.1)	30	87.6(29.3)	30	0.39
Exhaled nitric oxide, ppb		54.5(59.5)	17	31.6(21.0)	29	40.0(47.2)	30	0.11
Sputum culture								
	Aspergillus spp.(+), n (%)	4(12.9)	31	0	30	1(3.1)	32	0.06
	Fungus (+), n (%)	5(16.1)	31	0	23	1(3.1)	32	0.08
	P. aeruginosa (+), n (%)	7(22.6)	31	4(13.3)†	30	12(37.5)	32	0.08
	Gram negative bacteria (+), n (%)	9(29.0)	31	15(50.0)	30	17(53.1)	32	0.11
Airway lesions								
	mReiff score	4.6(4.6)	38	3.3(3.8)	35	3.4(3.7)	38	0.40
	Number of bronchiolitis affected lobes	2.5(2.0)	38	3.0(2.2)	35	3.0(2.3)	37	0.59
	Mucus score	3.6(3.7)	20	3.0(2.8)	21	4.0(3.6)	21	0.68

Data are shown mean (SD). P value in the last column demonstrates variance across the 3 groups.

*p<0.05 vs ABPA, † p<0.05 vs *Af* sIgE^{-} group. FEV_1, forced expiratory volume in one second.

Table E3. Univariate analysis for associations between previous mucus score and previous clinical indices in all patients

Variables	Rho (p value)
White blood cells	0.51（0.001）
Blood neutrophils	0.33（0.0503）
eosinophils	0.30（0.08）
monocytes	0.42（0.01）
FEV_1/FVC	-0.60（0.001）
$\%FEV_1$	-0.60（0.001）

TABLE E4. Present data for each cluster

		CL 1	N	CL 2	N	P
Blood test	White blood cell, /μL	8744(3085)	34	7033(3699)	20	0.02
	Neutrophil, /μL	5795(2823)	34	4867(3238)	20	0.07
	Monocyte, /μL	510(179)	34	378(148)	20	0.007
	Eosinophil, /μL	602(613)	34	300(503)	20	0.002
	Basophil, /μL	62(42)	34	51(93)	20	0.006
	Total IgE, IU/mL	1566(2654)	31	1494(1829)	16	0.65
FEV_1/Forced vital capacity, %		63.8(14.2)	24	69.5(13.3)	19	0.27
%FEV_1, %		69.6(27.3)	24	94.4(30.4)	19	0.004
Exhaled nitric oxide, ppb		30.5(16.9)	21	42.6(39.6)	15	0.46
Sputum culture						
	Aspergillus spp.(+), n (%)	0	30	2(11.1)	18	0.14
	Fungus (+), n (%)	1(3.3)	30	2(11.1)	18	0.55
	P. aeruginosa (+), n (%)	9(30.0)	30	1(5.6)	18	0.07
	Gram negative bacteria (+), n (%)	15(50.0)	30	5(27.8)	18	0.13
Airway lesions						
	mReiff score	4.6(4.8)	33	3.6(3.5)	21	0.68
	Number of bronchiolitis affected lobes	3.2(2.1)	33	2.2(2.1)	21	0.09
	Mucus score	4.8(3.4)	18	1.5(2.3)	16	0.002

Data are shown mean (SD). FEV_1, forced expiratory volume in one second.
